# Estrogen-secreting testicular tumors in 46,XY female patients with 17α-hydroxylase/17,20-lyase deficiency: two unusual case reports and a review of the literature

**DOI:** 10.3389/fgene.2025.1508792

**Published:** 2025-04-17

**Authors:** Julio Americo Pereira Batatinha, Mirian Yumie Nishi, Rafael Loch Batista, José Antônio Diniz Faria Júnior, Maria Helena Palma Sircili, Francisco Tibor Denes, Maria Jimena Chafloque Mesia, Laura da Silva Salvanini, Elaine Maria Frade Costa, Filomena Marino Carvalho, Berenice Bilharinho Mendonca, Sorahia Domenice

**Affiliations:** ^1^ Unidade de Endocrinologia do Desenvolvimento, Laboratório de Hormônios e Genética Molecular LIM/42, Hospital das Clínicas da Faculdade de Medicina da Universidade de São Paulo, São Paulo, Brazil; ^2^ Laboratório de Sequenciamento em Larga Escala (SELA), Faculdade de Medicina da Universidade de São Paulo, São Paulo, Brazil; ^3^ Departamento de Urologia do Hospital das Clínicas da Faculdade de Medicina da Universidade de São Paulo, São Paulo, Brazil; ^4^ Departamento de Patologia da Faculdade de Medicina da Universidade de São Paulo,São Paulo, Brazil

**Keywords:** 46,XY differences in sex development, 17α-hydroxylase/17, 20-lyase deficiency, *CYP17A1* variants, testicular tumor, seminoma, Leydig cell tumor

## Abstract

**Context:**

17α-hydroxylase/17,20-lyase deficiency (17OHD) is a rare autosomal recessive condition. Women who have the complete form of 17OHD typically have a female phenotype, with an absence of secondary sexual characteristics, primary amenorrhea, and hypertension, which is usually detected in adolescence. Generally, 46,XY patients with a partial form of 17OHD have atypical genitalia and intra-abdominal or inguinal testes. The risk of developing malignant testicular tumors or testicular adrenal rest tumors in 21-hydroxylase deficiency congenital adrenal hyperplasia is reported in 46,XY patients. In contrast, these conditions are rarely described in patients with 17OHD.

**Objective:**

This study aims to investigate patients with 17OHD who exhibit testicular tumors and spontaneous pubertal development.

**Patients and Results:**

Two unrelated women with 46,XY karyotype with 17OHD who presented with unexpected spontaneous development of secondary sexual characteristics and testicular tumors were described. Pathogenic allelic variants in *CYP17A1* were identified in the compound heterozygous state in both patients. The variants p.Trp406Arg and p.Pro428Leu were identified in the patient with Leydig cell neoplasia plus germ cell neoplasia *in situ*, and the p.Arg358Gln and p.Trp406Arg variants were identified in the patient with intratubular seminoma associated with invasive classic seminoma.

**Conclusion:**

Our findings reinforce the risk of testicular tumor development among 46,XY patients with 17OHD and add data to the discussion of the risk/benefit ratio of prophylactic gonadectomy in the treatment patients with 46, XY differences in sex development (DSD).

## Introduction

Differences of sex development (DSD) due to 17α-hydroxylase/17,20-lyase deficiency (17OHD) is a rare autosomal recessive disorder.

The P450c17 enzyme is a type 2 microsomal P450 enzyme that performs both steroid 17α-hydroxylase and 17,20-lyase activities. The *CYP17A1* gene, located on chromosome 10 (10q24.3), encodes the P450c17 enzyme (Miller, 2018). Both enzymes are involved in the steroid biosynthesis pathway by controlling cortisol and sex steroid production. A reduction in cortisol production due to 17-hydroxylase deficiency results in increased adrenocorticotropic hormone (ACTH) secretion and, consequently, increased production of 11-deoxycorticosterone, which has potent mineralocorticoid activity. Moreover, reducing androgen production by impairing 17,20-lyase activity leads to androgen and estrogen deficiencies (Auchus, 2001).

Differences in several clinical features were reported in patients with 17OHD based on whether activities of both enzymes are affected (i.e., 17α and 17,20-lyase) and the severity of enzyme dysfunction (i.e., whether residual activity is present). In addition, the karyotype of the affected individuals also impacts the phenotype (Marsh and Auchus, 2014).

17OHD can be classified mainly into complete or partial forms according to clinical features and biochemical and hormonal data. Rarely, isolated 17,20-lyase deficiency was also described (Maheshwari et al., 2022).

Individuals affected with 46,XX have female internal and external genitalia, mainly primary amenorrhea, and impairment of sexual maturation secondary to inadequate ovarian androgen and estrogen biosynthesis (Huang et al., 2017); for example, they may experience spontaneous development of breasts and pubic hair but have primary amenorrhea. Some may exhibit menarche but progress to oligomenorrhea or secondary amenorrhea (Marsh and Auchus, 2014). Recurrent ovarian cysts are frequently observed. High gonadotropin levels resulting from the lack of negative estrogen feedback are associated with ovarian cyst development (Ten Kate-Booij, 2004; Carvalho et al., 2016). Infertility is caused by folliculogenesis interruption and anovulation, in addition to chronically elevated levels of adrenal-derived progesterone (Turcu and Auchus, 2015). However, it is possible for patients with 17OHD deficiency to conceive with *in vitro* fertilization (Bianchi et al., 2016).

The low androgen production in 46,XY patients with 17OHD results in undervirilized external genitalia, which can be present as female-like or atypical genitalia. In the complete combined form of 17OHD, a phenotypically 46,XY female individual usually seeks healthcare due to primary amenorrhea and absence of secondary sex characteristics; these individuals present with female external genitalia and lack hypoplastic Wolffian duct derivatives. The testes may be identified in the intra-abdominal or inguinal region or at labioscrotal folds (Williams Textbook of Endocrinology). In 46,XY patients with partial forms of 17OHD, poorly virilized external genitalia are seen at birth, accompanied by poor virilization at puberty. Gynecomastia may be observed in patients with this condition, especially with residual enzyme activity (Maheshwari et al., 2022). Hypertension is common (Wu et al., 2017). The accumulation of mineralocorticoid precursors (deoxycorticosterone) causes low-renin hypertension and hypokalemic alkalosis (Takeda et al., 2001). However, normotension was reported in approximately 10%–15% of patients with 17OHD, regardless of the degree of loss of P450c17 enzyme activity (Wang et al., 2014). Affected 46,XY individuals with isolated 17,20-lyase deficiency exhibit low sex hormone secretion and atypical genitalia, normal glucocorticoid and mineralocorticoid secretion, and absence of low-renin hypertension (Miller, 2012).

Unlike other 46,XY DSD conditions, 17OHD patients are at low risk of malignant transformation of germ cells (Looijenga et al., 2010). Therefore, testicular tumors have been rarely reported in patients with 17OHD (Han et al., 2021). Here, we describe the cases of two unrelated women with 46,XY with 17OHD who presented with unexpected spontaneous secondary characteristics of sexual development (breasts and pubic hair) and Leydig cell proliferation associated with testicular tumors.

## Statement of ethics

This study was approved by the Ethical Committee of Hospital das Clinicas of the University of Sao Paulo Medical School, University of Sao Paulo. Written informed consent was obtained from both patients and their parents.

## Methods

Hormonal assay (IFMA, AutoDELFIA): dehydroepiandrosterone sulfate (DHEA-S), luteinizing hormone (LH), and follicle-stimulating hormone (FSH) were measured by electrochemiluminescence assay. Total testosterone (T), progesterone, and estradiol (E2) were measured by electrochemical immunoassay. 17-OH-Progesterone and androstenedione were measured by liquid chromatography with tandem mass spectrometry (LC‒MS-MS). Aldosterone was measured by the chemiluminescence assay. ACTH was measured by the immunochemiluminescence assay. Serum cortisol levels were measured by the immunochemical assay.

Both patients were administered a depot injection of a GnRH analog to ascertain whether the source of estrogen was adrenal or gonadal. Additionally, should the source be determined to be gonadal, testosterone levels may offer valuable insights into whether estrogen was synthesized within the gonads or aromatized peripherally. Blood samples were collected immediately before and 48 h after the administration of a 3.75-mg intramuscular injection of leuprorelin.

*Molecular studies*: Genomic DNA was obtained from peripheral blood leukocytes using the proteinase K and sodium dodecyl sulfate salting-out method. The *CYP17A1* gene (NM_000102.4/ENSG00000148795/ENST00000369887.4) coding region and the exon‒intron boundary areas were amplified by polymerase chain reaction (PCR). PCR products were purified using ExoSAP-IT (GE Healthcare Life Sciences), and sequencing was performed according to the protocol of the ABI Prism BigDye Terminator Ready Reaction Cycle Sequencing Kit (Life Technologies Corporation). The samples were submitted to the ABI Prism Genetic Analyzer 3130XL (Life Technologies Corporation). The sequences obtained were analyzed using Sequencher software (https://www.genecodes.com/). The allelic variants identified were classified according to the ACMG criteria using Franklin (https://franklin.genoox.com/clinical-db/home).

## Results

### Case presentation

#### Case 1

##### Patient information

A 33-year-old woman sought medical attention for primary amenorrhea. She had three younger sisters with the 46,XX karyotype with a 17OHD diagnosis and lack of spontaneous puberty or breast development before hormone treatment.

##### Clinical findings

She exhibited spontaneous development of breasts and pubic and axillary hair during her teenage years. Hypertension (150 × 100 mmHg), a height of 181 cm (Z score +3.2/target height Z score +0.1; female standards), a body mass index (BMI) of 25.9 kg/m^2^, Tanner stage 4 breast development, Tanner stage 5 pubic hair, and clitoromegaly (2.4 cm in length) were also observed.

##### Diagnostic assessment

A 46,XY karyotype was observed in the analysis of 20 cells. Biochemical analysis showed hypokalemia (K = 2.9 mEq/L), and the hormonal profile presented slightly high LH levels and normal E2 levels for the follicular phase, increased levels of FSH and progesterone, and low levels of T (Table 1, male reference values). ACTH levels were mildly elevated, and DHEA-S, cortisol, and aldosterone levels were low (Table 1). The pathogenic allelic variants c.1216T>C (p.Trp406Arg, rs104894143) and 1283C>T (p.Pro428Leu, rs104894145) in the *CYP17A1* gene in the compound heterozygous state were identified, confirming the diagnosis of 17OHD. Pelvic magnetic resonance imaging (MRI) confirmed the absence of a uterus and identified two pelvic gonads. The right gonad had a lobe-shaped solid lesion (5.2 × 4.0 × 3.0 cm, V- 32.5 cc). Serum was negative for the tumor biomarkers β-HCG, alpha-fetoprotein, carcinoembryonic antigen (CEA), and CA-125.

**TABLE 1:** Clinical–hormonal and genetic data of two 46,XY karyotype women with 17α-hydroxylase/17,20-lyase deficiency and testicular tumors.

Parameters	Case 1			Case 2		
Clinical characteristic						
Chronological age (yrs)	33			16		
Blood pressure (mmHg)	150 × 100			130 × 80		
Z-score height (male/female)	+1.02/+3.2			+2.52/+4.88		
BMI (m/kg^2^)	25.9			28.7		
Breast Tanner stage	4			4		
Pubic hair Tanner stage	5			5		
External genitalia	Female, clitoromegaly (length = 2.4 cm)			Female		

Hormonal profile						
Condition	Basal	24 h after GnRHa depot	Post gonadectomy	Basal	48 h after GnRHa depot	Post-gonadectomy
LH (1.7–8.6 IU/L)	9.4	132	34.6	8.4	67.4	31
FSH (3.5–12.5 IU/L)	16.8	116	33	8.1	15.6	81
E_2_ (<129 pmol/L)	154.14	267.91	<129	106.43	358.65	62.39
T (9.4–33.4 nmol/L)	0.42	0.42	-	0.62	0.42	0.42
Prog (<2.86 nmol/L)	66.78	104.94	5.72	4.45	13.99	2.23
17OHP (0.02–0.1 nmol/L)	0.04	0.07	-	0.47	-	0.03
Andro (<0.077 nmol/L)	0.017	0.017	-	0.01	-	0.01
DHEAS (2.68–9.23 μmol/L)	0.28	<0.21	-	0.98	-	0.29
ACTH (<9.9 pmol/L)	15.84	-	-	5.72	-	37.62
Cortisol (138–690 nmol/L)	55.2	-	-	295.32	-	248.4
Aldosterone (<0.64 nmol/L)	0.083	-	-	0.69	-	0.20

Genetic diagnosis		
*CYP17A1* variant	p.Trp406Arg/p.Pro428Leu	p.Arg358Gln/p.Trp406Arg

GnRHa depot, GnRH analog depot (3.75 mg); E_2_, estradiol; T, testosterone; ACTH, adrenocorticotropic hormone; Prog, progesterone; 17OHP, 17-OH progesterone; Andro, androstenedione; DHEAS, dehydroepiandrosterone sulfate; NL, normal range of male hormonal levels.

In addition, the patient hormonal study demonstrated an increased secretion of estradiol after a GnRH analog depot injection (basal levels: 42 pg/mL, levels 48 h after a GnRHa injection: 73 pg/mL) without an increase in the testosterone levels (12 ng/dL) (Table 1).

##### Therapeutic intervention

**FIGURE 1:**
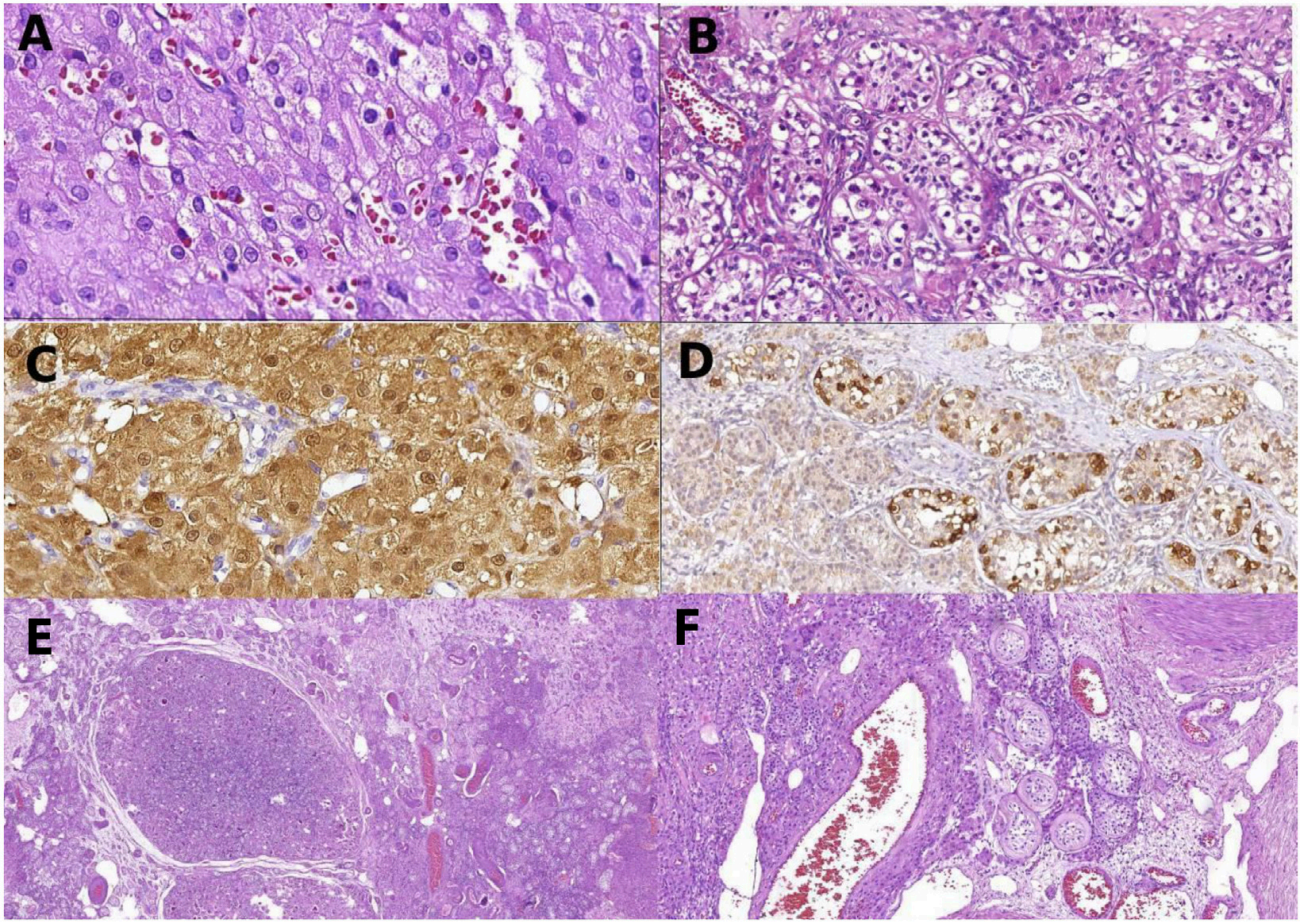
Histology of gonadal tissue of patient 1: Leydig cell tumor composed, a uniform population of Leydig cells **(A)**; germ cell neoplasia *in situ* with large atypical cells in seminiferous tubules **(B)**; Leydig cells of the right tumor, positive to calretinin by immunohistochemistry **(C)**; atypical germ cells of germ cell neoplasia *in situ* positive to PLAP **(D)**; panoramic view of the left gonad showing Sertoli nodules among testicular tissue **(E)**; non-neoplastic gonadal tissue of the right gonads with Leydig cell hyperplasia and seminiferous tubules with peritubular fibrosis, filled by Sertoli cells **(F)**.

The patient underwent a laparoscopic bilateral gonadectomy. The histopathological study identified Leydig cell neoplasia plus germ cell neoplasia *in situ* (GCNIS) in the right testis and GCNIS in the left testis (pathological staging: pT1 pNx pMx) (Figures 1A, B). The immunohistochemical analysis showed that the Leydig cell tumor was positive for calretinin and inhibin (alpha subunit), Sertoli cells were positive for CD-99, and GCNIS was positive for placental alkaline phosphatase and OCT-4 (Figures 1C, D). In normal peritumoral testicular parenchyma, different degrees of atrophy of the seminiferous tubules and peritubular fibrosis, Sertoli cell nodules, and exuberant Leydig cell hyperplasia were identified (Figures 1D–F). After gonadectomy, the E2 levels became undetectable (Table 1). The patient was treated with continuous oral estrogen, dexamethasone (0.25 mg daily), and anti-hypertensive medication (spironolactone and losartan potassium).

##### Follow-up and outcomes

No signal of tumor recurrence was observed in the 7-year follow-up period after the gonads were removed.

#### Case 2

##### Patient information

A 16-year-old woman sought medical attention for primary amenorrhea. No familial history of DSD conditions was reported.

##### Clinical findings

Thelarche occurred spontaneously at age 13, and thelarche occurred at age 14. Clinical examination showed normotension (130 × 80 mmHg), a height of 177.5 cm (Z score +2.55/target height Z score −0,45; female standards), a BMI of 28.7 kg/m2, and acanthosis nigricans. Tanner stage 4 breast development, Tanner stage 5 pubic hair, and typical female external genitalia were observed.

##### Diagnostic assessment

A 46,XY karyotype was observed in the analysis of 20 cells. The hormonal profile showed normal-to-high LH and FSH levels, normal E2 levels, increased progesterone and 17OHP levels, and low T levels (Table 1, male reference values). The levels of ACTH, DHEA-S, cortisol, and aldosterone were within the normal range (Table 1). Additionally, pathogenic allelic variants c.1073G>A (p.Arg358Gln, rs104894139) and c.1216T>C (p.Trp406Arg, rs104894143) were identified in a compound heterozygous state in the *CYP17A1* gene, confirming 17OHD diagnosis. Pelvic MRI showed the absence of a uterus and ovaries and identified two nodular structures suggestive of the testes; the right testis was located in the inguinal canal (3.5 × 2.0 cm), and the left testis was in the intra-abdominal position (2.9 × 1.5 cm).

In addition, the patient hormonal study demonstrated an increased secretion of estradiol after a GnRH analog depot injection (basal levels are 29 pg/mL, and levels 48 h after a GnRHa injection are 95 pg/mL) without an increase in testosterone levels (12 ng/dL) (Table 1).

##### Therapeutic intervention

Laparoscopic bilateral gonadectomy was performed, and no distinguishable macroscopic mass was identified. The histopathological study revealed similar findings in both gonads: intratubular seminoma associated with invasive classic seminoma. Adjacent non-neoplastic gonadal tissue presented Sertoli cell nodules, seminiferous tubules with Sertoli cells without signs of spermatogenesis, and Leydig cell hyperplasia (pathological staging: pT1 pNx pMx). Immunohistochemical staining showed that Leydig cells were positive for calretinin; Sertoli cells were positive for CD-99; and neoplastic germ cells were positive for placental alkaline phosphatase, CD117/KIT, and D2-40 (podoplanin). Isolated estrogen replacement therapy was initiated in combination with dexamethasone (0.25 mg daily).

##### Follow-up and outcomes

No sign of tumor recurrence was observed in the 10-year follow-up period after the surgical procedure, and her blood pressure remained within the normal range.

## Discussion

Individuals with 46,XY DSD are more prone to develop germ cell tumors, but the malignancy risk is highly heterogeneous among DSD conditions (Marsh and Auchus, 2014; Huang et al., 2017; Liu et al., 2014).

The overall incidence of gonadal germ cells tumor (GCTs) among 46,XY DSD patients is estimated to be 2.3% (Cools et al., 2009). The germ cell tumor risk is the lowest (<5%) in patients with defects in androgen action or synthesis, whereas the risk ranges from 15% to 60% in those with gonadal dysgenesis (Abacı et al., 2015).

Data on the gonadal malignancy risk for 46,XY patients with 17OHD are limited. However, in a cohort of 62 patients undergoing gonadectomy with a molecular diagnosis of 17OHD, the histological analysis of the testicular tissue did not find testicular neoplasia in any gonad, reinforcing the rarity of this condition (Maheshwari et al., 2022). Consequently, it has been suggested that 17OHD represents a low-risk condition Germ Cell Tumors (GCTs), with no definitive recommendations regarding prophylactic gonadectomy for affected patients.

However, in some countries where the diagnosis of 17OHD is more frequent, such as Brazil and China, the diagnosis of gonadal tumors has been reported in these patients (Han et al., 2021). In our cohort of 15 Brazilian 46,XY patients diagnosed with 17OHD who underwent bilateral gonadectomy, a gonadal tumor was identified in two unrelated patients (13%). To date, the cases of five 46,XY patients with 17OHD and testicular tumors have been reported (Table 3) (Huang et al., 2017; Han et al., 2021; Lu et al., 2022). All of them were Chinese patients in whom the 17OHD diagnosis was established post-puberty.

In a previous study regarding the gonadal tumor risk in 292 phenotypic female patients with the Y chromosome or Y-derived sequence, the authors identified pelvic testicular tumors in two out of 22 patients with 17OHD (9.1%) (Huang et al., 2017). The gonadal tumors were a dysgerminoma and a Sertoli cell tumor (Table 3) (Huang et al., 2017), diagnosed at 20 and 22 years of age, respectively. One patient did not present with spontaneous pubertal development, whereas this information was unavailable for the other patient (personal communication). Their gonads were in the pelvis or inguinal canals. Genetic investigations to confirm the diagnosis were not performed in these patients.

Han et al. described the cases of two Chinese 46,XY patients carrying pathogenic allelic variants in *CYP17A1*, in whom testicular tumors were found. One of the patients presented with gonadoblastoma, and the other presented with germ cell neoplasia *in situ*. Both patients were also diagnosed in adulthood (28 and 35 years of age), and neither showed pubertal development at the time of diagnosis. Their gonads were in the pelvis or inguinal canals (Table 2) (Han et al., 2021). In the fifth Chinese patient, a laparoscopic prophylactic bilateral gonadectomy was performed at 15 years of age, and a bilateral Leydig cell tumor was identified (Lu et al., 2022). A literature review identified 153 post-pubertal 46,XY patients with 17OHD resulting from homozygous or compound heterozygous pathogenic variants in the *CYP17A1* gene (Maheshwari et al., 2022; Han et al., 2021; Lu et al., 2022). Including the patients from the current study, testicular tumors were found in five individuals, resulting in a frequency rate of 3.2% (Maheshwari et al., 2022; Han et al., 2021; Lu et al., 2022). Although these findings do not establish the prevalence of gonadal tumors in patients with 17OHD, they confirmed an increased risk for the development of testicular tumors in this condition.

**TABLE 2:** Histological and immunohistochemical analyses of testicular tumor from two 46,XY Brazilian patients with 17α-hydroxylase/17,20-lyase deficiency.

Patient	Age at surgery (Years)	Gonad position		Tumor histology	Immunohistochemistry	Non-neoplastic gonadal tissue	Wolffian derivative
1	33	Pelvic	Right	Leydig cell tumor and GCNIS	Calretinin+ and inhibin (α-subunit)+	Sertoli cell nodules; seminiferous tubules with variable atrophy; Leydig cell hyperplasia	Rudimentary epididymis
			Left	GCNIS	PLAP+; OCT4+		
2	17	Inguinal	Right	Seminoma + ITS	PLAP+; OCT4+; CD117+; D2-40+		Epididymis
		Abdominal	Left				

GCNIS, germ cell neoplasia *in situ*; ITS, intratubular seminoma; PLAP, placental alkaline phosphatase; D2-40, Podoplanin.

Several factors may influence gonadal tumor development in patients with 46,XY DSD, such as the involvement of genes located on the Y chromosome, the location and histology of the gonads, and hormonal factors (Barros et al., 2021). In all 46,XY patients with 17OHD, it is possible to identify some predisposing risk factors for the development of a testicular tumor, such as the patient’s age at the time of the tumor diagnosis (15–33 years) and the presence of undescended testes (abdominal, pelvic, and inguinal testes).

The frequency of germ cell tumors in adult men with undescended testes, which may be located in the abdominal or pelvic cavity, is significantly higher than that in the general population. The risk is particularly elevated during adolescence and early adulthood. Likewise, the anatomical position of the gonads is a predictive factor for germ cell tumor risk in patients with 46,XY DSD.

Furthermore, the aberrant expression of embryonic and early differentiation markers [OCT3/4, cKIT, and placental alkaline phosphatase (PLAP)] in testicular tissue has been associated with the onset of preneoplastic lesions and further germ cell tumor development (Barros et al., 2021).

In all 46,XY patients with 17OHD, it is possible to identify some predisposing risk factors for the development of a testicular tumor, such as the patient’s age at the time of the tumor diagnosis (15–33 years) and the presence of undescended testes (abdominal, pelvic, and inguinal testes).

The anatomical position of the gonads is a predictive parameter of germ cell tumor risk. It is well established that the risk for gonadal tumor development is higher in an abdominal rather than a scrotal gonad in 46,XY patients with the same DSD condition (Looijenga et al., 2010; Barros et al., 2021). Moreover, there is a consensus that the risk of gonadal malignancy in patients with 46,XY DSD increases with age (Barros et al., 2021). Hence, it is hypothesized that sex steroid exposure, especially to androgens, during and after puberty stimulates the development of germ cell tumors in 46,XY DSD patients. This fact is well-demonstrated in patients with androgen insensitivity syndrome (AIS). Patients with the partial phenotype of AIS (PAIS) are at a higher risk of developing gonadal tumors than patients with the complete phenotype of AIS (CAIS) (Cools et al., 2005; Cools et al., 2006). A factor contributing to the higher risk of PAIS is the residual activity of the androgen receptor, which promotes germ cell survival (Cools and Looijenga, 2017; Kaprova-Pleskacova et al., 2014). However, in some conditions, such as 46,XY complete gonadal dysgenesis and 17OHD with low/absent residual enzymatic activity, the development of a gonadal tumor at pubertal age is observed even in the absence of testosterone, suggesting the involvement of other mechanisms. The incomplete gonadal differentiation is an important factor related to tumor development in XY dysgenetic gonads.

Germ cell and sex cord–stromal tumors were described in seven patients with 17OHD—two in our cases and five in the literature. GCNIS was identified in three patients [patients 1, 2, and 6], seminoma in two patients (patients 2 and 3) (Auchus, 2001; Marsh and Auchus, 2014), Leydig cell tumors in two (patients 1 and 7), Sertoli cell tumor in one (patient 4), and gonadoblastoma in one (patient 5) (Table 3) (Huang et al., 2017; Miller, 2012; Cools et al., 2009). Although germ cells and immature sex cord cells have distinct origins, both are associated with gonadoblastoma, a known precursor of germ cell neoplasia such as seminoma and GCNIS (Al-Obaidy and Idrees, 2021). The concomitance of germ cell neoplasia with sex cord–stromal tumors, as we found, is intriguing and hard to explain. We can hypothesize a possible role of undifferentiated gonadal tissue in cases of 17OHD.

**TABLE 3:** Description of clinical and histological data and residual enzymatic activities of the mutated P450c17 enzyme from seven 46,XY patients with 17α-hydroxylase/17,20-lyase deficiency and testicular tumors reported in the present study and in the literature.

Patient	External genitalia	Age at diagnosis	Gonad position	Spontaneous puberty	Tumor histology	Allelic variant	Enzyme activity		Reference
							17OH ase	17,20 lyase	
1	Clitoromegaly	33	Pelvic (R and L)	Yes	Leydig cell tumor (R) and GCNIS (R/L)	c.1216T>C, p.Trp406Arg (exon 8)	Null^1^	Null^1^	This study
						c.1283C>T, p.Pro428Leu (exon 8)	<5%^1^	Null^1^	
2	Female	17	Inguinal (R) and abdominal (L)	Yes	Seminoma (R/L) and GCNIS (R/L)	c.1073G>A, p.Arg358Gln (exon 7)	65%^2^	<5%^2^	This study
						c.1216T>C, p.Trp406Arg (exon 8)	Null^1^	Null^1^	
3	Female	20	Pelvic (R and L)	No	Seminoma	NA	NR	NR	Huang H. (2017)
4	Female	42	Pelvic (R and L)	ND	Sertoli cell tumor	NA	NR	NR	Huang H. (2017)
5	ND	28	Inguinal and pelvic	No	Gonadoblastoma (R)	c.1247G>A, p.Arg416His (exon 8)	Null^3^	Null^3^	Han et al. (2021)
						c.1427T>C, p.Leu476Pro (exon 8)	NR	NR	
6	ND	35	Inguinal (R and L)	No	GCNIS (R)	c.985_987delTACinsAA, p.Tyr329Lysfs (exon 6)	Null^1^	NR	Han et al. (2021)
						c.1306G>A, p.Gly436Arg (exon 8)	Null^4^	NR	
7	Female	15	NR	NR	Leydig cell tumor (R/L)	c.985_987delTACinsAA	Null^1^	NR	Lu et al. (2022)
						p.Tyr329Lysfs (exon 6)			

R, right; L, left; NA; not available; GCNIS, germ cell neoplasia *in situ*; NR, not reported in the literature; ND, not described.

1, Reference 29; 2, Reference 30; 3, Reference 32; 4, Reference 16.

Among immunomarkers of germ cells, OCT3/4 is a transcription factor essential for stem cell functions that aids in their identification in tumors such as germinomas (GCNIS, seminomas, and dysgerminomas) and gonadoblastoma, but it is reduced in more differentiated germ cell tumors, for example, yolk-sac tumors and teratomas (Cheng et al., 2004; Jones et al., 2004). Other germ cell immunohistochemical markers can be used to classify a germ cell component, for example, D2-40 (podoplanin), CD30, SALL4, CD117 (KIT), and glypican (Al-Obaidy and Idrees, 2021). Both GCNIS and seminoma are strongly positive for OCT3/4, SALL4, and CD117, while embryonal carcinoma express OCT3£ and CD30, and yolk-sac tumors are negative for OCT3/4 and positive for SALL4 and glypican (Al-Obaidy and Idrees, 2021). A particular challenge is the differential diagnosis of GCNIS and non-neoplastic seminiferous tubules with delayed maturation. In both conditions, germ cells are OCT3-/4-positive but irregularly distributed in GCNIS and located basally and centrally in delayed maturation. In turn, the sex cord–stromal component is negative for germ cell markers and positive for inhibin (alpha subunit), calretinin, WT1, Melan A, FOXL2, and steroid factor-1 (SF1), among others (Al-Obaidy and Idrees, 2021). We emphasize the need to use the immunohistochemical approach to classify the cell components of the gonads of DSD in order to identify a neoplastic/precursor condition, such as GCNIS and gonadoblastoma, as well as correctly classify a malignant neoplasm. The molecular diagnosis of CYP17A1 defects was confirmed in three of the five Chinese patients diagnosed with gonadal tumors described previously (Table 3). Two patients carried compound heterozygous variants, and one carried a variant in homozygosity (Table 3). Compound heterozygous variants were also identified in the Brazilian patients.

The activity assessment of P450c17 enzyme variants identified in the patients with testicular tumors revealed a severe enzymatic impairment and androgen production abolishment (Table 3) in all but one (p.Leu476Pro variant), for whom the information was not available. In addition, the *CYP17A1* variant c.1073G>A (p.Arg358Gln, rs104894139) in patient 2 severely compromised 17,20-lyase activity (Table 3) (Costa-Santos et al., 2004; Geller et al., 1999; DeVore and Scott, 2012; Ergun-Longmire et al., 2006).

In agreement with the expected severe enzymatic impairment, all patients had female external genitalia with mild signs of androgenic activity (mild clitoromegaly in patient 1). Interestingly, both Brazilian patients spontaneously developed breasts, suggesting a sex steroid action at pubertal age. As mentioned in the results section, their hormonal study demonstrated an increased secretion of estradiol after a GnRH analog depot injection (basal levels are 42 and 29 pg/mL, levels 48 h after a GnRHa injection are 73 and 95 pg/mL) without an increase in testosterone levels (12 ng/dL in both patients) (Table 1).

Therefore, we speculate that estrogen production in these two cases might be associated with the presence of the tumor tissue. The stimulated secretion of estradiol observed in the two patients, without a simultaneous increase in testosterone secretion, indicates the new ability of the tumoral cells to rapidly aromatize the produced testosterone to estradiol. To make this hormone condition possible, testicular neoplastic cells must express changes in the enzymatic machinery concerning the original gonadal cells, which carry the *CYP17A1* variants responsible for the patients’ disease. Unfortunately, we were unable to confirm this hypothesis.

## Conclusion

The occurrence of gonadal tumors in 46.XY individuals with 17OHD is rare; however, this risk should be taken into account when managing these patients. Furthermore, in 46,XY individuals with 17OHD, spontaneous breast development may also indicate the presence of gonadal tumors in this specific phenotype.

Our findings reinforce the risk of testicular tumor development among individuals with 46,XY DSD due to 17OHD, adding arguments to discuss the risk/benefit balance of prophylactic gonadectomy in the treatment decision-making approach for patients with this 46,XY DSD condition.

## Data Availability

The raw data supporting the conclusions of this article will be made available by the authors, without undue reservation.
